# Effectiveness and Safety of the TRIO Optimal Health Management Program in Patients With Type 2 Diabetes Mellitus Initiating Basal Insulin Therapy: Prospective Observational Real-World Study

**DOI:** 10.2196/67554

**Published:** 2025-01-13

**Authors:** Chenxi Li, Lixin Guo, Lixin Shi, Li Chen, Liming Chen, Yaoming Xue, Hong Li, Yuzhen Liang, Jing Yang, Weimin Wang, Dalong Zhu

**Affiliations:** 1 Department of Endocrinology, Endocrine and Metabolic Disease Medical Center Nanjing Drum Tower Hospital, Affiliated Hospital of Nanjing Medical School Nanjing China; 2 Department of Endocrinology Peking Union Medical College, Chinese Academy of Nanjing Medical Sciences Beijing China; 3 Department of Endocrinology Affiliated Hospital of Guiyang Medical University Guiyang China; 4 Department of Endocrinology Qilu Hospital of Shandong University Jinan China; 5 NHC Key Laboratory of Hormones and Development, Tianjin Key Laboratory of Metabolic Diseases, Chu Hsien-I Memorial Hospital & Tianjin Institute of Endocrinology Tianjin Medical University Tianjin China; 6 Department of Endocrinology & Metabolism Nanfang Hospital Southern Medical University Guangzhou China; 7 Department of Endocrinology and Metabolism The First Affiliated Hospital of Kunming Medical University Kunming China; 8 Department of Endocrinology The Second Affiliated Hospital of Guangxi Medical University Nanjing China; 9 Department of Endocrinology First Hospital of Shanxi Medical University Taiyuan China

**Keywords:** type 2 diabetes, TRIO optimal health management program, initiating basal insulin therapy, glycemic control, real-world study

## Abstract

**Background:**

Diabetes, a chronic disease necessitating long-term treatment and self-management, presents significant challenges for patients who spend most of their treatment time outside of hospitals. The potential of digital therapeutics for diabetes has garnered recognition from different organizations. Although some prior studies have demonstrated successful reductions in patients’ blood glucose levels and body weight through digital diabetes programs, many studies were limited by including patients with prediabetes, including patients treated with mostly premixed insulin, or evaluating user engagement outcomes rather than clinical outcomes. Consequently, limited evidence remains regarding the effectiveness of health management mobile apps specifically designed for patients with type 2 diabetes mellitus (T2DM) initiating basal insulin (BI). Based on this, a data-based and artificial intelligence management system named “TRIO” was developed to provide patients with more personalized intervention methods in stages, in groups, and around the clock. TRIO assists doctors and nurses in achieving better blood glucose controls, truly carries out standardized management around patients, and allows them to have a higher quality of life. TRIO represents the 3 essential pillars in comprehensive diabetes management: physician, nurse, and patient.

**Objective:**

This prospective observational study evaluated the effectiveness and safety of the TRIO optimal health management program for patients with T2DM initiating BI therapy in a real-world setting.

**Methods:**

Patients aged 18-85 years with inadequate glycemic control (baseline hemoglobin A_1c_ [HbA_1c_] ≥7%) starting BI therapy were enrolled in outpatient and inpatient settings. The study lasted 3 months, with health education and phone-based follow-up assessments. Data collected included patient characteristics, medical history, baseline diabetes conditions, treatment compliance, glycemic control, and safety indicators.

**Results:**

A total of 199,431 patients were included, and 118,134 patients completed the 3-month follow-up between December 1, 2019, and December 31, 2021, involving 574 hospitals in China. The mean baseline HbA_1c_ was 9.2%, the mean duration of diabetes was 7.3 years, and 80.4% (1,59,930/1,98,969) of patients were using BI with oral antihyperglycemic drugs. After the intervention, mean HbA_1c_ decreased by –2.59% from baseline, with 55.6% (28,858/51,912) achieving the target HbA_1c_ level of <7%. Patients who set lower fasting plasma glucose goals (<6.1 mmol/L) showed more significant HbA_1c_ reductions (*P*<.001) and higher target achievement than those with fasting plasma glucose goals of ≥6.1 mmol/L. Factors such as complications, diabetes duration, and baseline HbA_1c_ levels influenced the magnitude of HbA_1c_ reduction. The presence of complications, shorter diabetes duration, and higher baseline HbA_1c_ were significantly associated with increased hypoglycemia incidence risk (all *P*<.05).

**Conclusions:**

The TRIO optimal health management program effectively improved glycemic control in patients with T2DM initiating BI therapy. Individualized treatment approaches considering patient characteristics and glycemic goals are vital for optimal outcomes.

## Introduction

### Background

The prevalence of type 2 diabetes mellitus (T2DM) in China is rapidly increasing due to lifestyle changes and an aging population. As per the 2018 American Diabetes Association criteria, the estimated prevalence of total diabetes and prediabetes among Chinese adults escalated to 12.8% and 35.2%, respectively, between 2015 and 2017 [1]. Despite the wide range of medication options available for antidiabetic treatment, glycemic control rates among patients with T2DM remain suboptimal [2]. A national survey conducted in 2018 revealed that only 32.9% (10,071/30,609) of patients with diabetes received treatment, with only half of them (15,336/30,609, 50.1%) achieving adequate glycemic control [3].

Diabetes, a chronic disease necessitating long-term treatment and self-management, presents significant challenges for patients who spend most of their treatment time outside of hospitals [4]. When lifestyle intervention and oral antidiabetic drugs (OADs) fail to provide optimal control, patients with type 2 diabetes are required to initiate injectable therapies, mostly basal insulin (BI), according to 2020 Chinese guidelines for T2DM management [5].

Previous randomized controlled trials (RCTs) and observational studies have shown the efficacy of BI in controlled trials [6] and real-world settings [7]. Maintaining a delicate equilibrium between achieving optimal blood glucose control and mitigating hypoglycemia risks is pivotal. This involves the appropriate titration of insulin and diligent self-monitoring of blood glucose levels, both of which are integral to sustaining effective glycemic management. Consequently, establishing an optimal diabetes management framework encompassing health education, consistent professional follow-up, and comprehensive self-monitoring tools becomes imperative for effectively managing patients with type 2 diabetes [8]. The lack of comprehensive and patient-centered approaches in current health care systems further compounds the burden of diabetes management. Time constraints and resource availability often limit traditional health education and face-to-face interactions with health care providers. As a result, there is a growing need for innovative solutions to bridge these gaps and provide ongoing support to individuals with type 2 diabetes [9-11]. The emergence of digital tools such as mobile apps and WeChat miniprograms has increased application in diverse therapeutic domains, such as attention-deficit/hyperactivity disorder, cancer, asthma, and insomnia tools to augment patient self-management [12].

### Objectives

The potential of digital therapeutics for diabetes has garnered recognition from different organizations, such as the Centers for Disease Control and Prevention and the Digital Therapeutics Alliance [13]. Although some prior studies have demonstrated successful reductions in patients’ blood glucose levels and body weight through digital diabetes programs up to a hemoglobin A_1c_ (HbA_1c_) reduction of 0.49% [14], many studies were limited by including patients with prediabetes [15], including patients treated with mostly premixed insulin [16], or evaluating user engagement outcomes rather than clinical outcomes [17]. Consequently, limited evidence remains regarding the effectiveness of health management mobile apps specifically designed for patients with T2DM initiating BI.

Accordingly, we have developed a personalized health management program combined with artificial intelligence named “TRIO” for initiating BI therapy in patients with type 2 diabetes and aim to evaluate its effectiveness and safety. Unlike conventional acronyms or abbreviations, TRIO does not represent a longer phrase but rather represents the 3 essential pillars in comprehensive diabetes management: physician, nurse, and patient. This program combines traditional health education with a mobile app to enhance diabetes management. Through this evaluation, we aspire to contribute to understanding practical approaches to optimizing glycemic control and promoting patient well-being.

## Methods

### Study Design and Population

**Figure 1:**
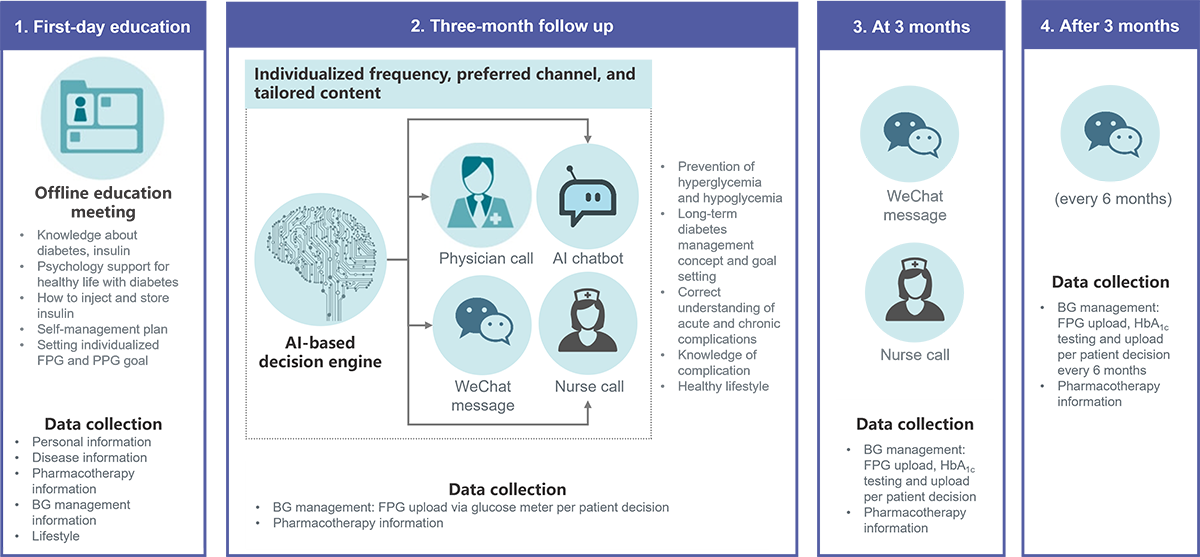
The operating process of the TRIO optimal health management program. AI: artificial intelligence; BG: blood glucose; FPG: fasting plasma glucose; HbA_1c_: hemoglobin A1c; PPG: postprandial glucose.

This prospective, 3-month observational program aimed to evaluate the effectiveness and safety of TRIO, an optimal health management program for patients with T2DM initiating BI therapy in a real-world setting. Participants were recruited from outpatient departments and, at discharge, from inpatient departments between December 1, 2019, and December 31, 2021, from 594 hospitals in China. Patients were assessed for their suitability by the following criteria: (1) aged between 18 and 85 years; (2) patients with T2DM who were inadequately controlled by OADs at the time of enrollment (ie, baseline HbA_1c_ level of ≥7%); (3) initiating BI therapy during the program period, meaning they had not used BI within 12 weeks prior to enrollment; (4) absence of mental disorders or communication impairments; and (5) absence of severe illnesses or limitations regarding follow-up. Patients who fulfilled these eligibility requirements were enrolled upon their willingness to provide informed consent. On the first day of enrollment, patients received health education from nurses, including knowledge about diabetes and insulin, psychological support for a healthy life with diabetes, and how to inject and store insulin. In addition, the physicians assisted patients in drawing up a self-management plan and helped them set individualized fasting plasma glucose (FPG) and postprandial glucose (PPG) goals. Patients were also asked to follow the WeChat official account of the TRIO program, through which knowledge about diabetes management would be sent. Follow-up assessments were conducted via phone calls at 1, 2, 4, 8, and 12 weeks. The frequency and follow-up methods were tailored to each patient’s FPG level. If the FPG level was 7 mmol/L, a phone call was not scheduled for the next follow-up visit, and only a WeChat message was sent (Figure 1).

### Ethical Considerations

This study was approved by the ethics committee of Nanjing Drum Tower Hospital (institutional review board review approval document, code: 2019-231-01), which was the principal research institute representing other subcenters. Implied consent was obtained from all participants when they registered on the TRIO WeChat official account following the principles of the Declaration of Helsinki, as the privacy policy included a clause allowing anonymized data to be used for research purposes. Participant privacy and anonymity were achieved through the elimination of any patient identifiers such as name or dates of birth, which were anonymized and deidentified before extraction and securely stored in compliance with data protection regulations. No compensation was provided to participants, as this study involved a secondary analysis of existing data. No identifiable images of participants were included in the study or supplementary materials, eliminating the need for additional image consent.

### Data Collection

Baseline information was collected by interviews at the hospital enrollment, including demographics, disease characteristics, medical history, physical examination, BI types, starting dosage, and concomitant antidiabetic drugs (bolus insulin, glucagon-like peptide-1 receptor agonists, or OAD) used with BI. Laboratory tests including HbA_1c_ and FPG were obtained in hospitals at baseline, while glycemic control regarding HbA_1c_ and self-monitoring blood glucose (SMBG), including fasting blood glucose (FBG), dosage, and hypoglycemia information during follow-up time, were self-reported. Self-reported data were collected through phone calls by nurses or uploaded via a smart blood glucose device or input into the TRIO WeChat official account by the patients.

### Outcomes

- *Primary effectiveness end points*: The primary effectiveness end point of our analysis is the change in HbA_1c_ levels from baseline to month 3.
- *Secondary effectiveness end points*: In addition to the primary end point, we also examined various other measures related to glycemic control. These secondary end points include changes in FBG levels from baseline to month 3, the achievement of target HbA_1c_levels (<7%), the achievement of target FBG levels (<7 and <6.1 mmol/L), and an assessment of changes in BI dose.
- *Safety end points*: Our safety end points include monitoring and assessing the incidence and rates of hypoglycemia events and evaluating composite end points. The 3-month end point included the incidence and rates of hypoglycemia events and a composite end point encompassing the percentage of patients reaching target HbA_1c_ and FBG levels without experiencing hypoglycemia events.

### Statistical Methods

Continuous variables were described using mean and SD, while categorical variables were presented as frequencies and percentages. For continuous effectiveness indicators, a paired *t* test (2-tailed) was applied to test the significance of the change from baseline to month 3 in HbA_1c_ or FPG in the total population. Analysis of covariance was used to compare these changes between subgroups, including patient source (inpatient or outpatient), complication status (no or yes), duration of diabetes (<5 years or ≥5 years), baseline HbA_1c_ (7%-8%, 8%-9%, 9%-10%, or ≥10%), and FBG goal setting (≥6.1 mmol/L or <6.1 mmol/L). Least square (LS) mean (SE) and LS mean difference with 95% CIs were provided. For binary effectiveness outcomes (HbA_1c_ <7%, FBG <7 mmol/L, or FBG <6.1 mmol/L), multivariable logistic regression models were applied to explore the association of subgroups with outcomes and variables included in the model were the same as analysis of covariance model. Hypoglycemic incidence and rate were evaluated by SMBG, uploaded by the smart glucose blood device or manual input to the TRIO platform by patients. Hypoglycemic incidence (percentage of patients with SMBG ≤3.9 mmol/L or SMBG <3.0 mmol/L) was analyzed using logistic regression, and odds ratio (OR) with 95% CI was used as the effect size; hypoglycemic rate (numbers of events per patient-year) was investigated by Poisson regression, and risk ratio (RR) with 95% CI were used as the effect size for this analysis. Composite end points including HbA_1c_ <7% without SMBG ≤3.9 mmol/L, FBG <7 mmol/L without SMBG ≤3.9 mmol/L, and FBG <6.1 mmol/L without SMBG ≤3.9 mmol/L were also explored using logistic regression. All the analyses were conducted using SAS 9.4 (SAS Institute, Inc), and a 2-sided *P* value of <.05 was considered statistically significant.

## Results

### Participant Recruitment

**Figure 2:**
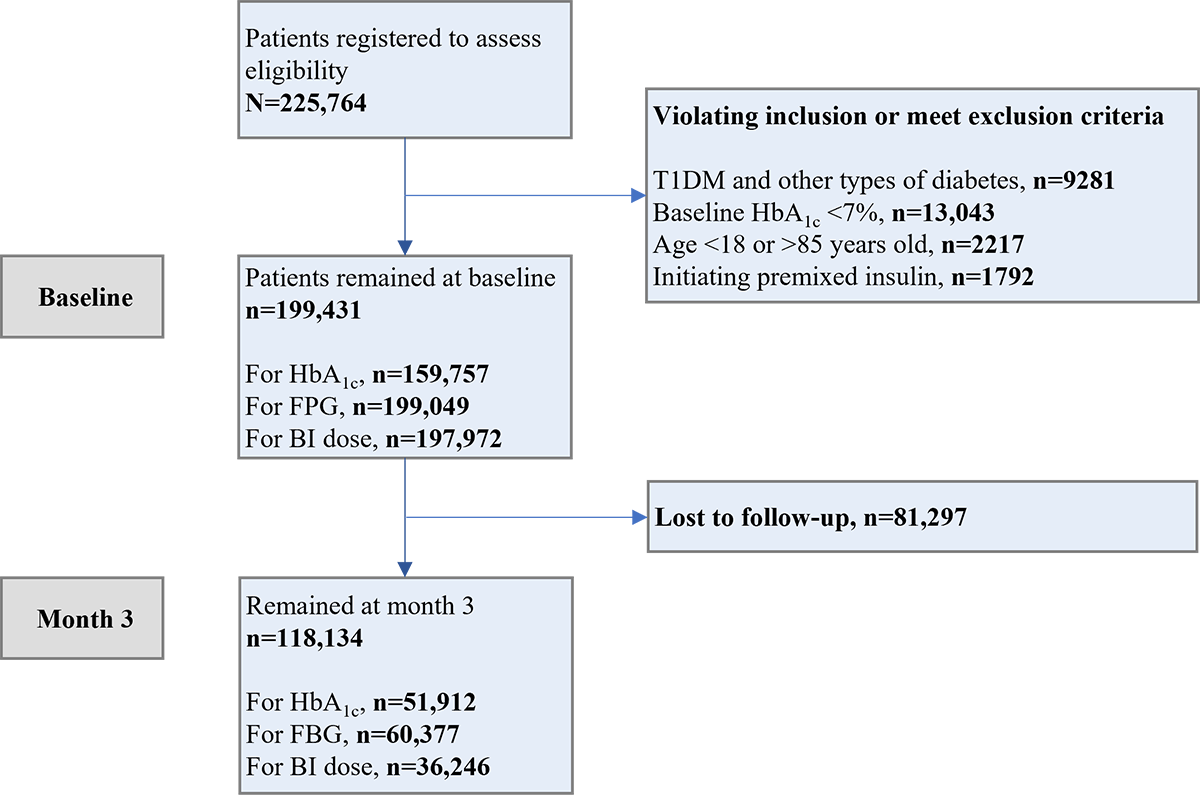
Flowchart of participating patients enrolled in the TRIO optimal health management program. Nested values under "Patients remained at baseline" and "Remained at month 3" are non-mutually exclusive. BI: basal insulin; FPG: fasting plasma glucose; HbA_1c_: hemoglobin A1c; T1DM: type 1 diabetes mellitus.

Between December 1, 2019, and December 31, 2021, a total of 225,764 patients were recruited from 594 hospitals. A total of 26,333 patients were excluded because of violating inclusion or meeting exclusion criteria, leaving 199,431 patients remaining at baseline. Among them, 81,297 patients were lost to follow-up within the first 3 months, resulting in 118,134 patients who completed the 3-month follow-up with measurements of either HbA_1c_ or FPG (Figure 2).

### Baseline Characteristics

The patients’ mean (SD) age at baseline was 57.3 (12.5) years, with 42.8% (85,337/1,99,431) of participants being women. The average BMI was 24.8 kg/m^2^. At baseline, the mean HbA_1c_, FPG, and PPG levels were 9.6%, 9.5 mmol/L, and 12.7 mmol/L, respectively. The mean duration of diabetes was 7.3 years. The most common complications or comorbidities observed in our patient cohort were hypertension (55,637/1,96,023, 28.4%), hyperlipidemia (32,240/1,96,023, 16.4%), and peripheral neuropathy (63,145/1,96,023, 32.2%). Most patients were taking BI with oral antihyperglycemic drugs (OADs) (1,59,930/1,98,969, 80.4%), with some also using prandial insulin concurrently.

Outpatients had a slightly higher mean age of 57.7 (SD 12.2) years than inpatients, with a mean age of 57.0 (SD 12.6) years. Gender distribution revealed that 56.3% (39,802/70,704) of outpatients were male, while 57.6% (74,064/1,28,645) of inpatients were male. Moreover, the duration of diabetes was slightly longer in inpatients, with a mean of 7.4 years (SD 6.8) than in outpatients, with a mean of 7.0 years (SD 6.3). Both groups displayed a similar average BMI of 24.8 kg/m^2^ and exhibited comparable values for blood pressure and lipid levels such as triglycerides, total cholesterol, low-density lipoprotein, and baseline HbA_1c_. Meanwhile, baseline FPG (10.3 vs 9.0 mmol/L) and PPG (14.0 vs 12.2 mmol/L) were slightly higher in outpatients than in inpatients. A notable difference was observed in the percentage of patients with complications or comorbidities, with 31.3% (39,950/1,27,610) of inpatients having hypertension compared with 22.9% (15,687/68,413) of outpatients and 38.1% (48,580/1,27,610) of inpatients having peripheral neuropathy compared with 21.3% (14,565/68,413) of outpatients. Inpatients also had a higher proportion of patients being treated with BI in combination with prandial insulin, accounting for 20.3% (26,037/1,28,342) of inpatients as opposed to 15.5% (10,924/70,627) of outpatients (Table 1). No clinically significant differences in baseline characteristics were observed between patients who remained in the study at month 3 and those who were lost to follow-up (Table S1 in Multimedia Appendix 1).

**Table 1:** Baseline characteristics of patients enrolled in the TRIO optimal health management program.

Baseline characteristics			Outpatient (n=70,786)		Inpatient (n=128,645)		All (N=199,431)
**Age (years), mean (SD)**			57.7 (12.2)		57.0 (12.6)		57.3 (12.5)
**Sex, n (%)**							
	Male	39,802 (56.3)		74,064 (57.6)		1,13,866 (57.2)	
	Female	30,902 (43.7)		54,435 (42.4)		85,337 (42.8)	
**BMI (kg/m^2^), mean (SD)**			24.8 (3.3)		24.8 (3.5)		24.8 (3.5)
**Duration of diabetes (years), mean (SD)**			7.0 (6.3)		7.4 (6.8)		7.3 (6.6)
**SBP^a^ (mm Hg), mean (SD)**			131.8 (15.4)		131.8 (16.5)		131.8 (16.2)
**DBP^b^ (mm Hg), mean (SD)**			79.9 (10.1)		79.6 (10.4)		79.7 (10.3)
**Triglycerides (mmol/L), mean (SD)**			2.3 (2.0)		2.3 (2.1)		2.3 (2.1)
**Total cholesterol (mmol/L), mean (SD)**			4.6 (1.5)		4.7 (1.5)		4.7 (1.5)
**LDL^c^ (mmol/L), mean (SD)**			2.8 (1.1)		2.8 (1.1)		2.8 (1.1)
**BI^d^ dose (U/d), mean (SD)**			15.2 (5.5)		16.2 (6.1)		15.9 (5.9)
**BI dose (U/kg/d), mean (SD)**			0.23 (0.08)		0.24 (0.09)		0.24 (0.09)
**eGFR^e^ (mL/min/1.73 m^2^), mean (SD)**							
	<90	953 (23.6)		3999 (22.0)		4952 (22.3)	
	90-120	1135 (28.2)		4738 (26.1)		5873 (26.5)	
	≥120	1942 (48.2)		9424 (51.9)		11,366 (51.2)	
**Baseline HbA_1c_^f^ (%), mean (SD)**			9.3 (1.8)		9.7 (2.1)		9.6 (2.0)
**Baseline FPG^g^ (mmol/L), mean (SD)**			10.3 (3.3)		9.0 (3.2)		9.5 (3.3)
**Baseline PPG^h^ (mmol/L), mean (SD)**			14.0 (4.4)		12.2 (4.0)		12.7 (4.2)
**Regimen, n (%)**							
	BI alone ± OAD^i^	59,176 (83.8)		1,00,754 (78.5)		1,59,930 (80.4)	
	BI + prandial insulin ± OAD	10,924 (15.5)		26,037 (20.3)		36,961 (18.6)	
	BI + GLP-1 RA^j^ ± OAD	527 (0.7)		1551 (1.2)		2078 (1.0)	
**Comorbidity, n (%)**							
	Hypertension	15,687 (22.9)		39,950 (31.3)		55,637 (28.4)	
	Hyperlipemia	8992 (13.1)		23,248 (18.2)		32,240 (16.4)	
	Left ventricular hypertrophy	53 (0.08)		149 (0.12)		202 (0.10)	
	Atrial fibrillation	80 (0.12)		213 (0.17)		293 (0.15)	
**Complication, n (%)**							
	Stroke	1409 (2.1)		4511 (3.5)		5920 (3.0)	
	Coronary heart disease	4601 (6.7)		11,656 (9.1)		16,527 (8.3)	
	Diabetic nephropathy	3266 (4.8)		11,209 (8.8)		14,475 (7.4)	
	Diabetic retinopathy	6058 (8.9)		17,329 (13.6)		23,387 (11.9)	
	Diabetic foot	663 (1.0)		3253 (2.6)		3916 (2.0)	
	Peripheral neuropathy	14,565 (21.3)		48,580 (38.1)		63,145 (32.2)	
	Lower extremity angiopathy	2844 (4.2)		10,601 (8.3)		13,445 (6.9)	

^a^SBP: systolic blood pressure.

^b^DBP: diastolic systolic blood pressure.

^c^LDL: low-density lipoprotein.

^d^BI: basal insulin.

^e^eGFR: estimated glomerular filtration rate.

^f^HbA_1c_: hemoglobin A_1c_.

^g^FPG: fasting plasma glucose.

^h^PPG: postprandial glucose.

^i^OAD: oral antidiabetic drug.

^j^GLP-1 RA: glucagon-like peptide-1 receptor agonists.

### Initial Regimens and Adherence to BI Treatment

During the intervention, the majority of patients initiated insulin glargine (1,77,331/1,98,969, 89.1%) as their primary treatment, while a smaller proportion started with insulin determir (6588/1,98,969, 3.3%), neutral protamine Hagedorn insulin (766/1,98,969, 0.4%), or insulin degludec (8939/1,98,969, 4.5%). Alongside BI treatment, 41% (57,242/1,39,739), 28.2% (39,473/1,39,739), and 9.7% (13,545/1,39,739) of patients were concurrently taking 1, 2, and ≥3 OADs, respectively. The most commonly used OADs were metformin (69,027/1,39,739, 49.4%) and α-glucosidase inhibitors (46,960/1,39,739, 33.6%), followed by sodium-glucose cotransporter-2 inhibitors (22,911/1,39,739, 16.4%), dipeptidyl peptidase-4 inhibitors (20,722/1,39,739, 14.8%), sulfonylureas (7266/1,39,739, 5.2%), glinides (6257/1,39,739, 4.5%), and thiazolidinediones (5504/1,39,739, 3.9%). Sulfonylureas were more frequently prescribed to outpatients (3571/48,696, 7.3%) than inpatients (3695/91,043, 4.1%), while sodium-glucose cotransporter-2 inhibitors were more commonly used in inpatients (16,615/91,043, 18.2%) than outpatients (6296/48,696, 12.9%; Table S2 in Multimedia Appendix 1).

### Glycemic Outcomes

At the end of the 3-month management intervention period, the mean HbA_1c_ level among 44,847 participants with eligible self-reported mean HbA_1c_ measurements was 6.89% (SD 0.90). This represented a mean decrease in HbA_1c_ by –2.59% (SE 0.01; *P*<.001) from baseline (Table 2). Regarding FBG levels, at the end of month 3, the mean FBG level among 60,365 participants with eligible self-reported FBG measurements was 6.81 (SD 1.4) mmol/L, indicating an average decrease in FBG by –2.77 (SD 0.01) mmol/L (*P*<.001) from baseline (Table 2 and Figure S1 in Multimedia Appendix 1). For the HbA_1c_ target, 55.6% (28,858/51,912) of participants achieved the target HbA_1c_ level of <7% after the 3-month intervention period (Table 3). Similarly, 61.3% (37,017/60,377) and 29.2% (17,633/60,377) of participants reached FBG levels of <7.0 and <6.1 mmol/L, respectively, at the end of the 3-month intervention period.

In the subgroup analyses, patients with complications experienced slightly lower HbA_1c_ (LS mean difference: 0.07, 95% CI 0.05-0.09) than those with no complications. Considering the duration of diabetes, patients with a duration of ≥5 years exhibited a lesser decrease in HbA_1c_ (LS mean difference: 0.09, 95% CI 0.07-0.11) than those with a duration of <5 years. A higher baseline HbA_1c_ level was also associated with a more significant reduction. Compared with patients with baseline HbA_1c_ in the range of 7% to 8%, patients with a baseline HbA_1c_ in the range of 8% to 9% had the lesser decrease (LS mean difference: –0.83, 95% CI –0.86 to –0.79), while patients with a baseline HbA_1c_ of 10% or higher had the highest decrease (LS mean difference: –4.04, 95% CI –4.08 to –4.00). Also, patients with initial FBG goal setting of <6.1 mmol/L had a greater decrease in their HbA_1c_ (LS mean difference: –0.36, 95% CI –0.38 to –0.34) than those with FBG goal setting of ≥6.1 mmol/L (Table 2). In the subgroup analysis exploring factors related to FBG reduction, consistent results with HbA_1c_ reductions found that more significant reductions were seen in patients with no complications, diabetes duration of <5 years, and an initial FBG goal setting of <6.1 mmol/L, except for baseline HbA_1c_. Patients with lower baseline HbA_1c_ exhibited higher reductions in FBG from baseline (Table 2).

**Table 2:** HbA_1c_^a^ and fasting blood glucose change from baseline to month 3 after initiation of basal insulin therapy with TRIO monitoring.

				Values, n		Baseline, mean (SD)		Month 3, mean (SD)		LS^b^ mean (SE)	*P* value		LS mean difference (95% CI)		*P* value
**HbA_1c_ change from baseline to month 3**															
	**All**		44,847		9.48 (1.98)		6.89 (0.90)		–2.59 (0.01)		<.001	N/A^c^			
	**Patient sources**														
		Outpatient	14,893		9.10 (1.71)		6.93 (0.81)		–2.63 (0.02)		<.001	Reference			
		Inpatient	29,954		9.67 (2.07)		6.88 (0.95)		–2.71 (0.01)		<.001	–0.08 (–0.1 to –0.06)		<.001	
	**Complication**														
		No	17,963		9.33 (1.89)		6.83 (0.85)		–2.71 (0.02)		<.001	Reference			
		Yes	20,389		9.58 (2.01)		6.96 (0.93)		–2.64 (0.01)		<.001	0.07 (0.05 to 0.09)		<.001	
	**Duration (years)**														
		<5	18,950		9.69 (2.14)		6.80 (0.88)		–2.72 (0.02)		<.001	Reference			
		≥5	25,888		9.32 (1.83)		6.96 (0.92)		–2.63 (0.01)		<.001	0.09 (0.07 to 0.11)		<.001	
	**Baseline HbA_1c_ (%)**														
		7-8	9250		7.42 (0.33)		6.78 (0.88)		–0.79 (0.02)		<.001	Reference			
		8-9	12,485		8.34 (0.32)		6.86 (0.89)		–1.62 (0.02)		<.001	–0.83 (–0.86 to –0.79)		<.001	
		9-10	8342		9.35 (0.31)		6.94 (0.86)		–2.54 (0.01)		<.001	–1.75 (–1.79 to –1.70)		<.001	
		≥10	14,770		11.81 (1.57)		6.97 (0.95)		–4.83 (0.02)		<.001	–4.04 (–4.08 to –4.00)		<.001	
	**FBG^d^ goal setting (mmol/L)**														
		≥6.1	36,935		9.54 (1.98)		6.96 (0.87)		–2.49 (0.01)		<.001	Reference			
		<6.1	7904		9.22 (1.96)		6.58 (0.99)		–2.85 (0.02)		<.001	–0.36 (–0.38 to –0.34)		<.001	
**FBG change from baseline to month 3**															
	**All**		60,365		9.58 (3.30)		6.81 (1.40)		–2.77 (0.01)		<.001	N/A			
	**Patient sources**														
		Outpatient	21,055		10.35 (3.27)		6.86 (1.39)		–2.61 (0.02)		<.001	Reference			
		Inpatient	39,310		9.17 (3.25)		6.79 (1.41)		–2.68 (0.02)		<.001	–0.07 (–0.09 to –0.04)		<.001	
	**Complication**														
		No	22,927		9.86 (3.16)		6.68 (1.32)		–2.71 (0.02)		<.001	Reference			
		Yes	28,252		9.14 (3.17)		6.94 (1.45)		–2.58 (0.02)		<.001	0.13 (0.11 to 0.16)		<.001	
	**Duration (years)**														
		<5	24,683		9.74 (3.54)		6.57 (1.27)		–2.78 (0.02)		<.001	Reference			
		≥5	35,666		9.48 (3.13)		6.98 (1.46)		–2.50 (0.02)		<.001	0.28 (0.25 to 0.3)		<.001	
	**Baseline HbA_1c_ (%)**														
		7-8	10,005		8.35 (2.13)		6.68 (1.26)		–2.75 (0.02)		<.001	Reference			
		8-9	13,472		9.21 (2.48)		6.79 (1.29)		–2.66 (0.02)		<.001	0.08 (0.05 to 0.12)		<.001	
		9-10	9506		9.59 (2.99)		6.85 (1.39)		–2.61 (0.02)		<.001	0.13 (0.09 to 0.17)		<.001	
		≥10	18,879		10.32 (4.11)		6.83 (1.49)		–2.58 (0.01)		<.001	0.16 (0.13 to 0.20)		<.001	
	**FBG goal setting (mmol/L)**														
		≥6.1	50,482		9.68 (3.32)		6.87 (1.40)		–2.52 (0.02)		<.001	Reference			
		<6.1	9854		9.10 (3.18)		6.53 (1.35)		–2.76 (0.02)		<.001	–0.24 (–0.28 to –0.21)		<.001	

^a^HbA_1c_: hemoglobin A_1c_.

^b^LS: least squares.

^c^N/A: not applicable.

^d^FBG: fasting blood glucose.

**Table 3:** Target HbA_1c_^a^ and fasting blood glucose at month 3 after initiation of basal insulin therapy with TRIO monitoring.

						Values, n			Month 3, n (%)			OR^b^ (95% CI)			*P* value
**HbA_1c_ (<7% at month 3)**															
	**All**				51,912			28,858 (55.6)			N/A^c^				
	**Patient sources**														
		Outpatient		18,753			10,024 (53.5)			Reference					
		Inpatient		33,159			18,834 (56.8)			1.16 (1.11-1.21)			<.001		
	**Complication**														
		No		20,881			12,433 (59.5)			Reference					
		Yes		23,008			12,016 (52.2)			0.85 (0.81-0.89)			<.001		
	**Duration (years)**														
		<5		21,873			13,388 (61.2)			Reference					
		≥5		30,030			15,466 (51.5)			0.83 (0.79-0.87)			<.001		
	**Baseline HbA_1c_ (%)**														
		7-8		9250			5573 (60.2)			Reference					
		8-9		12,485			6974 (55.9)			0.85 (0.8-0.9)			<.001		
		9-10		8342			4455 (53.4)			0.77 (0.72-0.83)			<.001		
		≥10		14,770			8009 (54.2)			0.72 (0.68-0.77)			<.001		
	**FBG^d^ goal setting (mmol/L)**														
		≥6.1		43,051			22,857 (53.1)			Reference					
		<6.1		8845			5993 (67.8)			1.8 (1.7-1.9)			<.001		
**FBG (<7 mmol/L at month 3)**															
	**All**				60,377			37,017 (61.3)			N/A				
	**Patient sources**														
		Outpatient		21,065			12,403 (58.9)			Reference					
		Inpatient		39,312			24,614 (62.6)			1.17 (1.12-1.22)			<.001		
	**Complication**														
		No		22,936			14,952 (65.2)			Reference					
		Yes		28,254			16,239 (57.5)			0.84 (0.8-0.88)			<.001		
	**Duration (years)**														
		<5		24,686			17,053 (69.1)			Reference					
		≥5		35,675			19,955 (55.9)			0.70 (0.67-0.73)			<.001		
	**Baseline HbA_1c_ (%)**														
		7-8		10,006			6607 (66.0)			Reference					
		8-9		13,473			8288 (61.5)			0.84 (0.79-0.89)			<.001		
		9-10		9509			5710 (60.0)			0.79 (0.74-0.85)			<.001		
		≥10		18,881			11,632 (61.6)			0.77 (0.73-0.82)			<.001		
	**FBG goal setting (mmol/L)**														
		≥6.1		50,493			30,112 (59.6)			Reference					
		<6.1		9855			6886 (69.9)			1.42 (1.34-1.5)			<.001		
**FBG (<6.1 mmol/L at month 3)**															
	**All**				60,377			17,633 (29.2)			N/A				
	**Patient sources**														
		Outpatient		21,065			5763 (27.4)			Reference					
		Inpatient		39,312			11,870 (30.2)			1.16 (1.1-1.22)			<.001		
	**Complication**														
		No		22,936			7542 (32.9)			Reference					
		Yes		28,254			7087 (25.1)			0.83 (0.79-0.87)			<.001		
	**Duration (years)**														
		<5		24,686			8973 (36.3)			Reference					
		≥5		35,675			8656 (24.3)			0.7 (0.66-0.73)			<.001		
	**Baseline HbA_1c_ (%)**														
		7-8		10,006			3167 (31.7)			Reference					
		8-9		13,473			3764 (27.9)			0.84 (0.79-0.9)			<.001		
		9-10		9509			2625 (27.6)			0.87 (0.81-0.93)			<.001		
		≥10		18,881			5746 (30.4)			0.91 (0.85-0.96)			.002		
	**FBG goal setting (mmol/L)**														
			≥6.1	50,493			13,538 (26.8)			Reference					
			<6.1	9855			4084 (41.4)			1.7 (1.62-1.8)			<.001		

^a^HbA_1c_: hemoglobin A_1c_.

^b^OR: odds ratio.

^c^N/A: not applicable.

^d^FBG: fasting blood glucose.

Regarding target HbA_1c_ of <7% at month 3, patients enrolled from inpatient showed a slight advantage over outpatient (OR 1.16, 95% CI 1.11-1.21; *P*<.001), and patients with complications had lower odds of achieving HbA_1c_ target than those with no complications (OR 0.85, 95% CI 0.81-0.89; *P*<.001). Those with ≥5 years of duration of diabetes had lower success rates than those with <5 years of duration (OR 0.83, 95% CI 0.79-0.87; *P*<.001). Lower baseline HbA_1c_ levels were associated with better outcomes. Moreover, setting FBG target goal level of <6.1 mmol/L at the beginning of the treatment demonstrated a higher possibility of reaching the HbA_1c_ target of <7% at month 3 (OR 1.8, 95% CI 1.7-1.9; *P*<.001) (Table 3). Regarding the FBG target of <7 and <6.1 mmol/L at month 3, patients exhibited consistent results as in HbA_1c_ target of <7%. Inpatients; patients with no complications; and patients with diabetes duration of 5 years, lower HbA_1c_ target, and initial FBG goal setting of <6.1 mmol/L were associated with higher odds of achieving the target (Table 3).

### Insulin Dose and Satisfaction

Total insulin dose (U/d/kg) change was –0.01 (SD 0.06), from baseline (mean 0.23, SD 0.09) to month 3 (mean 0.22, SD 0.09; Table 4). Patients recruited from inpatients, with complications, having diabetes duration of ≥5 years, with FBG goal setting of <6.1 mmol/L and higher baseline HbA_1c_, had a higher starting dose of BI. Among 36,037 patients with both baseline and 3-month BI dosages, 7% (2546/36,037) remained unchanged, and 50.1% (18,047/36,037) lowered the dosage per kilogram (Table 4). Possible reasons for the lack of titration, such as patients reaching FBG targets or experiencing hypoglycemic events, were explored in Table S3 in Multimedia Appendix 1. Patients with stable or decreasing dosage during the 3 months had higher starting doses, higher percentages of FBG <7 mmol/L, and hypoglycemic incidence at week 1, 2, 4, 8, and 12 than patients with increasing dosage. Patient satisfaction level for TRIO was stable during the study. Furthermore, 99.6% (35,738/35,897) of the patients felt satisfactory or very satisfactory at month 3, and only 0.4% (159/35,897) chose average or below.

**Table 4:** Basal insulin dose (U/kg) change from baseline to month 3 by patient sources, with or with no complication, duration, baseline HbA_1c_^a^ levels, and target fasting plasma glucose levels.

		Values, n	Baseline, mean (SD)	Month 3, mean (SD)	Change, mean (SD)
**All**		36,037	0.23 (0.09)	0.22 (0.09)	–0.01 (0.06)
**Patient sources**					
	Outpatient	12,999	0.22 (0.08)	0.22 (0.08)	0.00 (0.05)
	Inpatient	23,038	0.24 (0.09)	0.23 (0.09)	–0.01 (0.06)
**Complication**					
	No	17,903	0.23 (0.08)	0.22 (0.08)	–0.01 (0.06)
	Yes	17,163	0.24 (0.09)	0.23 (0.09)	–0.01 (0.06)
**Duration (years)**					
	<5	13,993	0.22 (0.08)	0.21 (0.09)	–0.01 (0.06)
	≥5	22,034	0.24 (0.09)	0.23 (0.09)	0.00 (0.05)
**Baseline HbA_1c_ (%)**					
	7-8	5783	0.22 (0.08)	0.22 (0.08)	0.00 (0.05)
	8-9	8157	0.23 (0.09)	0.23 (0.09)	–0.01 (0.05)
	9-10	5711	0.23 (0.09)	0.22 (0.09)	–0.01 (0.05)
	≥10	11,003	0.24 (0.09)	0.23 (0.09)	–0.02 (0.06)
**FBG^b^ goal setting (mmol/L)**					
	≥6.1	30,034	0.23 (0.09)	0.22 (0.09)	–0.01 (0.06)
	<6.1	5992	0.24 (0.09)	0.23 (0.09)	–0.01 (0.06)
**Dose adjustment**					
	Up	15,444	0.22 (0.08)	0.24 (0.08)	0.02 (0.04)
	Keep	2546	0.23 (0.08)	0.23 (0.08)	0.00 (0.00)
	Down	18,047	0.25 (0.09)	0.21 (0.09)	–0.04 (0.06)

^a^HbA_1c_: hemoglobin A_1c_.

^b^FBG: fasting blood glucose.

### Incidence and Event Rate of Hypoglycemia

Hypoglycemia incidence (≤3.9 mmol/L) in all patients was 27.1% (3317/12,227). Inpatients had a higher incidence than outpatients (OR 1.24, 95% CI 1.07-1.45; *P*=.005). Patients with complications experienced more hypoglycemia (OR 1.25, 95% CI 1.07-1.45; *P*=.005). Longer diabetes duration (≥5 years) was associated with lower hypoglycemia incidence (OR 0.60, 95% CI 0.52-0.69; *P*<.001; Table 5). Higher baseline HbA_1c_ levels correlated with increased hypoglycemia risk. HbA_1c_ ≥10% had the highest incidence (OR 1.47, 95% CI 1.23-1.76; *P*<.001; Table 5). For hypoglycemic events defined by SMBG levels of ≤3.9 mmol/L, a total of 7619 events occurred, yielding an event rate of 2.49 events per person-year (Table 6). Notable trends included higher hypoglycemia rates for inpatient sources compared with outpatient sources (RR 1.25, 95% CI 1.08-1.46; *P*=.004), higher rates in patients with complications compared with those with no complications (RR 1.25, 95% CI 1.07-1.47; *P*=.006), and a lower rate in patients with a diabetes duration of ≥5 years (RR=0.67, 95% CI 0.59-0.77; *P*<.001). Furthermore, elevated baseline HbA_1c_ levels (≥10%) were associated with a higher hypoglycemia rate (RR 1.37, 95% CI 1.15-1.63; *P*=.001). An FBG goal setting of <6.1 before initiating BI was not associated with increased hypoglycemic incidence (OR 0.97, 95% CI 0.77-1.22; *P*=.79) or rate (RR 0.92, 95% CI 0.76-1.1; *P*=.35). For hypoglycemia defined as SMBG <3.0 mmol/L, 15.1% (1852/12,227) of patients with 2954 events were recorded, resulting in an incidence rate of 0.97 events per person-year. Similar trends were observed in relation to complications, duration of diabetes, and baseline HbA_1c_ levels. An FBG goal of <6.1 was not related to increased incidence (OR 0.97, 95% CI 0.77-1.22; *P*=.79) or rate (RR 1.01, 95% CI 0.80-1.27; *P*=.95) of hypoglycemia (Table 6).

**Table 5:** Hypoglycemia incidence during 3 months of TRIO monitoring in patients with self-monitoring blood glucose.

			Values, n		Month 3, n (%)		OR^a^ (95% CI)		*P* value
**SMBG^b^ (≤3.9 mmol/L)**									
	**All**		12,227		3317 (27.1)		N/A^c^		
	**Patient sources**								
		Outpatient	4581	1177 (25.7)		Reference			
		Inpatient	7646	2140 (28.0)		1.24 (1.07-1.45)		.005	
	**Complication**								
		No	1962	469 (23.9)		Reference			
		Yes	5125	1350 (26.3)		1.25 (1.07-1.46)		.005	
	**Duration (years)**								
		<5	5518	1803 (32.7)		Reference			
		≥5	6709	1514 (22.6)		0.60 (0.52-0.69)		<.001	
	**Baseline HbA_1c_^d^ (%)**								
		7-8	1953	390 (20.0)		Reference			
		8-9	2138	512 (23.9)		1.14 (0.94-1.38)		.20	
		9-10	1787	461 (25.8)		1.26 (1.03-1.55)		.02	
		≥10	3924	1276 (32.5)		1.47 (1.23-1.76)		<.001	
	**FBG^e^ goal setting (mmol/L)**								
		≥6.1	10,422	2827 (27.1)		Reference			
		<6.1	1777	484 (27.2)		0.98 (0.81-1.17)		.79	
**SMBG (<3.0 mmol/L)**									
	**All**		12,227		1852 (15.2)		N/A		
	**Patient sources**								
		Outpatient	4581	702 (15.3)		Reference			
		Inpatient	7646	1150 (15.0)		1.05 (0.87-1.26)		.64	
	**Complication**								
		No	1962	249 (12.7)		Reference			
		Yes	5125	753 (14.7)		1.26 (1.04-1.54)		.02	
	**Duration (years)**								
		<5	5518	1009 (18.3)		Reference			
		≥5	6709	843 (12.6)		0.63 (0.53-0.74)		<.001	
	**Baseline HbA_1c_ (%)**								
		7-8	1953	207 (10.6)		Reference			
		8-9	2138	289 (13.5)		1.24 (0.97-1.6)		.09	
		9-10	1787	259 (14.5)		1.34 (1.03-1.74)		.03	
		≥10	3924	727 (18.5)		1.64 (1.3-2.07)		<.001	
	**FBG goal setting (mmol/L)**								
		≥6.1	10,422	1584 (15.2)		Reference			
		<6.1	1777	262 (14.7)		0.97 (0.77-1.22)		.79	

^a^OR: odds ratio.

^b^SMBG: self-monitoring blood glucose.

^c^N/A: not applicable.

^d^HbA_1c_: hemoglobin A_1c_.

^e^FBG: fasting blood glucose.

**Table 6:** Hypoglycemia rates during 3 months of TRIO monitoring in patients with self-monitoring blood glucose.

				Events, n		Events/person-year		RR^a^ (95% CI)	*P* value
**SMBG^b^ (≤3.9 mmol/L)**									
	**All**		7619		2.49		N/A^c^		
	**Patient sources**								
		Outpatient	2581		2.25		Reference		
		Inpatient	5038		2.64		1.25 (1.08-1.46)		.004
	**Complication**								
		No	960		1.96		Reference		
		Yes	2897		2.26		1.25 (1.07-1.47)		.006
	**Duration (years)**								
		<5	4482		3.25		Reference		
		≥5	3137		1.87		0.67 (0.59-0.77)		<.001
	**Baseline HbA_1c_^d^ (%)**								
		7-8	871		1.78		Reference		
		8-9	1098		2.05		1.00 (0.82-1.21)		.97
		9-10	994		2.22		1.15 (0.94-1.4)		.18
		≥10	3168		3.23		1.37 (1.15-1.63)		.001
	**FBG^e^ goal setting (mmol/L)**								
		≥6.1	6449		2.48		Reference		
		<6.1	1155		2.60		0.92 (0.76-1.1)		.35
**SMBG (<3.0 mmol/L)**									
	**All**		2954		0.97		N/A		
	**Patient sources**								
		Outpatient	1138		0.99		Reference		
		Inpatient	1816		0.95		1.06 (0.87-1.29)		.56
	**Complication**								
		No	366		0.75		Reference		
		Yes	1156		0.90		1.24 (1.01-1.52)		.04
	**Duration (years)**								
		<5	1691		1.23		Reference		
		≥5	1263		0.75		0.66 (0.56-0.79)		<.001
	**Baseline HbA_1c_ (%)**								
		7-8	298		0.61		Reference		
		8-9	456		0.85		1.34 (1.04-1.72)		.03
		9-10	381		0.85		1.30 (0.99-1.70)		.06
		≥10	1253		1.28		1.72 (1.36-2.18)		<.001
	**FBG goal setting (mmol/L)**								
		≥6.1	2499		0.96		Reference		
		<6.1	447		1.01		1.01 (0.80-1.27)		.95

^a^RR: relative risk.

^b^SMBG: self-monitoring blood glucose.

^c^N/A: not applicable.

^d^HbA_1c_: hemoglobin A_1c_.

^e^FBG: fasting blood glucose.

Table 7 shows the composite end points of patients reaching the target with no hypoglycemia events during a 3-month period. For HbA_1c_ <7% without SMBG ≤3.9 mmol/L at month 3, patients with complications (OR 0.68, 95% CI 0.54-0.84; *P*=.001) and those with a duration of diabetes for ≥5 years (OR 0.76, 95% CI 0.62-0.93; *P*=.008) had significantly lower odds of reaching the target with no hypoglycemia events. Furthermore, higher baseline HbA_1c_ levels in the ranges of 9%-10% (OR 1.26, 95% CI 1.03-1.55; *P*=.02) and ≥10% (OR 1.47, 95% CI 1.23-1.76; *P*<.001) were associated with increased odds of achieving the target. Also, those who set an FBG goal of <6.1 mmol/L at initiation had significantly higher odds of reaching the composite end point (OR 1.35, 95% CI 1.03-1.79; *P*=.03). None of these factors were related to the composite end points for achieving FBG <7 mmol/L without SMBG ≤3.9 mmol/L at month 3. Finally, for achieving FBG <6.1 mmol/L without SMBG ≤3.9 mmol/L at month 3, only the duration of diabetes for ≥5 years was associated with a significantly lower possibility of achieving this composite end point.

**Table 7:** Composite end points of patients reaching target with no hypoglycemia events during 3 months of TRIO monitoring in patients with self-monitoring blood glucose.

			Values, n	Month 3, n (%)	OR^a^ (95% CI)	*P* value
**HbA_1c_^b^ <7% without SMBG^c^ ≤3.9 mmol/L**						
	**All**		5121	2055 (40.1)	N/A^d^	
	**Patient sources**					
		Outpatient	1987	750 (37.7)	Reference	
		Inpatient	3134	1305 (41.6)	1.14 (0.92-1.41)	.23
	**Complication**					
		No	735	352 (47.9)	Reference	
		Yes	1952	697 (35.7)	0.68 (0.54-0.84)	.001
	**Duration (years)**					
		<5	2325	1024 (44.0)	Reference	
		≥5	2796	1031 (36.9)	0.76 (0.62-0.93)	.008
	**Baseline HbA_1c_ (%)**					
		7-8	942	436 (46.3)	Reference	
		8-9	881	319 (36.2)	1.14 (0.94-1.38)	.20
		9-10	811	297 (36.6)	1.26 (1.03-1.55)	.02
		≥10	1495	579 (38.7)	1.47 (1.23-1.76)	<.001
	**FBG^e^ goal setting, mmol/L**					
		≥6.1	4435	1735 (39.1)	Reference	
		<6.1	686	320 (46.6)	1.35 (1.03-1.79)	.03
**FBG <7 mmol/L without SMBG ≤3.9 mmol/L**						
	**All**		5988	2398 (40.0)	N/A	
	**Patient sources**					
		Outpatient	2203	883 (40.1)	Reference	
		Inpatient	3785	1515 (40.0)	0.98 (0.81-1.18)	.80
	**Complication**					
		No	830	363 (43.7)	Reference	
		Yes	2531	947 (37.4)	0.85 (0.7-1.04)	.11
	**Duration (years)**					
		<5	2831	1197 (42.3)	Reference	
		≥5	3157	1201 (38.0)	0.93 (0.78-1.11)	.41
	**Baseline HbA_1c_ (%)**					
		7-8	957	438 (45.8)	Reference	
		8-9	1005	436 (43.4)	1.08 (0.85-1.37)	.53
		9-10	897	326 (36.3)	0.94 (0.73-1.20)	.61
		≥10	2050	807 (39.4)	0.94 (0.75-1.17)	.58
	**FBG goal setting, mmol/L**					
		≥6.1	5071	1989 (39.2)	Reference	
		<6.1	905	406 (44.9)	1.06 (0.84-1.33)	.62
**FBG <6.1 mmol/L without SMBG ≤3.9 mmol/L**						
	**All**		5988	1174 (19.6)	N/A	
	**Patient sources**					
		Outpatient	2203	438 (19.9)	Reference	
		Inpatient	3785	736 (19.4)	0.83 (0.66-1.04)	.11
	**Complication**					
		No	830	194 (23.4)	Reference	
		Yes	2531	434 (17.1)	0.81 (0.64-1.03)	.09
	**Duration (years)**					
		<5	2831	643 (22.7)	Reference	
		≥5	3157	531 (16.8)	0.74 (0.59-0.92)	.007
	**Baseline HbA_1c_ (%)**					
		7-8	957	213 (22.3)	Reference	
		8-9	1005	198 (19.7)	1 (0.75-1.34)	10
		9-10	897	139 (15.5)	0.79 (0.58-1.09)	.15
		≥10	2050	439 (21.4)	1.03 (0.79-1.36)	.82
	**FBG goal setting, mmol/L**					
		≥6.1	5071	955 (18.8)	Reference	
		<6.1	905	218 (24.1)	0.97 (0.74-1.29)	.86

^a^OR: odds ratio.

^b^HbA_1c_: hemoglobin A_1c_.

^c^SMBG: self-monitoring blood glucose.

^d^N/A: not applicable.

^e^FBG: fasting blood glucose

## Discussion

### Principal Findings

The TRIO program, a large-scale health management initiative using a digital WeChat platform for patients with T2DM initiating BI treatment, demonstrated its effectiveness in improving glycemic control 3 months after initiating BI. Prior to enrollment in TRIO, patients with T2DM exhibited suboptimal blood glucose control, with elevated baseline HbA_1c_ (9.6%) and FPG (9.5 mmol/L), a high prevalence of diabetic complications, and long diabetes duration (7.3 years). Following the 3-month TRIO management intervention, notable reductions in HbA_1c_ (–2.59%) and FBG (–2.77 mmol/L) were observed in the total population, accompanied by heightened proportions of achieving HbA_1c_ <7% (55.6%) and FBG target <7.0 mmol/L (61.3%) across diverse subgroups, such as patients from inpatient or outpatient care, patients with or with no complications, and patients with different length of diabetes duration, baseline HbA_1c_, and FBG goal setting. This study also highlights the potential for setting a lower FBG target (<6.1 mmol/L) at the initiation of BI compared with the traditional <7.0 mmol/L target. By setting a more rigorous FBG target of <6.1 mmol/L, better glycemic control was achieved without increased risk of hypoglycemia. These results hold promise for digital health tools such as TRIO in improving the overall management of T2DM in real-world clinical settings.

TRIO has shown effectiveness and safety in patients with T2DM initiating BI after OAD failure with the assistance of WeChat digital platform, which is consistent with previous single-arm studies incorporating digital tools conducted in patients with prediabetes [15,18] and patients treated with premixed insulin and BI [14,19,20]. For instance, the Omada Health Program investigated digital Diabetes Prevention Program engagement among patients with prediabetes, showing a reduction of –0.33 mmol/L in HbA_1c_ levels over 3 years [15]. In our TRIO study, with a larger sample size, we achieved a greater HbA_1c_ reduction of –2.58 mmol/L. Furthermore, in a 12-week German trial involving individuals with type 2 diabetes on BI, a smartphone app (My Dose Coach) was compared with a written titration chart. The intervention group using the app exhibited a noteworthy reduction in HbA_1c_ levels compared with the control group (–0.31%; *P*=.04), with safety outcomes remaining unaffected. These findings suggest that app-assisted titration can enhance glycemic control in patients with type 2 diabetes who use BI [20].

TRIO has demonstrated that digital tools including health education and self-management modules added on BI might provide additional benefits to effectiveness in glycemic control than medication alone. The ORBIT study is an observational registry conducted in China with patients with T2DM who were inadequately controlled by OADs and initiated BI [21], with similar baseline HbA_1c_ (9.6%, SD 2%) but higher baseline FBG (11.7, SD 4.0 mmol/L) and shorter diabetes duration (6.4, SD 5.3 years) than those in our study. Notably, the change in HbA_1c_ from baseline to month 3 demonstrated a more improvement in the TRIO group (–2.59%) than in the ORBIT group (–2%), as well as the attainment of the HbA_1c_ target of <7% at month 3 (55.6% vs 35.9%). Despite the relatively lower reduction in FBG levels in the TRIO study due to lower baseline FBG levels, a larger proportion of TRIO patients successfully reached the FBG target of <7 mmol/L (37,017/60,377, 61.3%) than those in the ORBIT study (2078/5571, 37.3%). Another significant study in this field, the First Basal Insulin Evaluation (FINE) Asia study, was a multinational, prospective, observational approach to assess BI’s efficacy in patients with uncontrolled type 2 diabetes (HbA_1c_ ≥8%) [22].

In TRIO, baseline HbA_1c_ and FBG were as high as 9.6% and 9.5 mmol/L, respectively (Table 1), which suggests delayed initiation of BI. The American Diabetes Association and the European Association for the Study of Diabetes suggest that BI should be promptly considered after the apparent “failure” of lifestyle modifications, including diet and exercise in combination with metformin, particularly when HbA_1c_ levels remain at or exceed 7% for a span of 2-3 months [23]. However, consistent with our findings, delay in injectable therapies was universal [24,25]. Timely initiation of insulin such as BI after the failure of oral treatment is associated with better glycemic control [26].

Standard T2DM management advice recommends keeping HbA_1c_ levels below 7%, but the ideal FPG target for achieving this is debated [27]. Different guidelines suggest varying FPG targets, such as 4.4-7.2 mmol/L according to the American Diabetes Association 2018 guidelines [28] or <6.1 mmol/L according to the American Association of Clinical Endocrinology-American College of Endocrinology and the International Diabetes Federation [29]. Previous studies support an FPG target of 6.1 mmol/L, showing better outcomes. Patients with FPG goals below 6.1 mmol/L had more significant HbA_1c_ reductions and higher target achievement rates without an increase in hypoglycemia [30,31]. Our results might confirm a better FPG target of <6.1 mmol/L. Patients who had an initial FPG goal setting below 6.1 mmol/L by their physician at the time of enrollment experienced both greater reductions in their HbA_1c_ levels (–2.64 vs –2.57%) and a higher HbA_1c_ target rate (67.8% vs 53.1%) than those with FPG goal setting ≥6.1 mmol/L (Tables 2 and 3). At the same time, hypoglycemic incidence and rate were comparable between the 2 groups. To enhance the management of hypoglycemic events, we recommend frequent SMBG monitoring, particularly during the initial weeks of insulin titration, to detect and address hypoglycemia promptly. Patient education on recognizing and treating hypoglycemic symptoms should be prioritized, alongside individualized insulin dose adjustments based on SMBG trends to minimize risk. Regular follow-ups are essential to reassess glycemic targets, such as FBG and HbA_1c_, and to prevent overtreatment while maintaining optimal glycemic control. These measures can help balance achieving strict glycemic targets with ensuring patient safety.

Regarding the titration of BI treatment, the current Chinese guideline recommends an initial dose of 0.2 U/kg or 10 U, underscoring the importance of active insulin dose adjustment to achieve optimal glycemic control [32]. Previous studies such as ORBIT have indicated inadequate titration, evident from a starting dose of 0.18 IU/kg/d and a final dose of 0.21 IU/kg/d, resulting in a change of +0.034 IU/kg/d. Within our program, comprehensive titration was not uniformly accomplished. Among the 36,037 patients with baseline and 3-month dosage data, 42.9% (15,444/36,037) of patients escalated their dosage during the program, while 7.1% (2546/36,037) maintained stability and 50.1% (18,047/36,037) of patients decreased their dosage. Consequently, there was a marginal numerical decline in dose by –0.01 (0.06) U/kg. Plausible explanations for this trend encompass the higher-than-recommended starting dose in our program, which even surpassed the final doses in earlier ORBIT studies. Furthermore, the pursuit of targeted FBG levels in the initial weeks and an increased incidence of hypoglycemic events among patients (as shown in Table S3 in Multimedia Appendix 1) could have contributed to these patterns.

### Limitations

Our study supports TRIO’s effectiveness and safety as a personalized health program, yet several limitations warrant acknowledgment. First, a significant portion of patients lacked HbA_1c_ follow-up data at month 3, possibly introducing a compliance bias that could overstate TRIO’s effectiveness by excluding those with less favorable HbA_1c_ reductions. Nonetheless, comparable baseline characteristics between compliant and noncompliant patients mitigate the risk of overestimation. Long-term data (months 3-12) are insufficient, leaving uncertainty about TRIO' s enduring effectiveness and ability to sustain patient adherence. Second, the judgment of hypoglycemia relied on SMBG, and the SMBG data were collected mainly through self-reporting on the WeChat platform, smart glucose devices, and regular phone calls by nurses, which may have a certain degree of deviation from the actual occurrence of hypoglycemia. Moreover, the absence of an RCT design and an external control group that included patients with type 2 diabetes who initiated only BI therapy without using TRIO may lead to the effect of TRIO to optimize glycemic control not solid enough. Therefore, it is necessary for future studies to design RCTs to evaluate the effectiveness and safety of intelligent blood glucose management in patients with diabetes with initiated BI therapy.

### Conclusions

The TRIO program has demonstrated effectiveness in glycemic control, as reflected in HbA_1c_ and FBG levels, among patients with T2DM initiating BI therapy. The program has improved HbA_1c_ and FBG target rates and patient compliance with insulin treatments. However, it is important to acknowledge the limitations of our study, including compliance bias, insufficient long-term follow-up data, and the need for further investigation using rigorous study designs. Future research, such as RCTs, is warranted to validate our study’s findings and assess TRIO’ s generalizability in real-world populations.

## Appendix

Multimedia Appendix 1 Loss to follow-up, dosage regimen, fasting blood glucose (FBG) target rate, incidence of hypoglycemia, and levels of FBG during the TRIO optimal health management program.

## Abbreviations

BI: basal insulin
FBG: fasting blood glucose
FPG: fasting plasma glucose
HbA_1c_: hemoglobin A1c
LS: least square
OAD: oral antidiabetic drug
OR: odds ratio
PPG: postprandial glucose
RCT: randomized controlled trial
RR: relative risk
SMBG: self-monitoring blood glucose
T2DM: type 2 diabetes mellitus

## Data Availability

The datasets generated during or analyzed during this study are not publicly available due to business confidentiality reasons but are available from the corresponding author upon reasonable request.

## References

[1] Li Y, Teng D, Shi X, Qin G, Qin Y, Quan H, Shi B, Sun H, Ba J, Chen B, Du J, He L, Lai X, Li Y, Chi H, Liao E, Liu C, Liu L, Tang X, Tong N, Wang G, Zhang J, Wang Y, Xue Y, Yan L, Yang J, Yang L, Yao Y, Ye Z, Zhang Q, Zhang L, Zhu J, Zhu M, Ning G, Mu Y, Zhao J, Teng W, Shan Z (2020). Prevalence of diabetes recorded in mainland China using 2018 diagnostic criteria from the American Diabetes Association: national cross sectional study. BMJ.

[2] Ramachandran A, Jain SM, Mukherjee S, Phatak S, Pitale S, Singh SK, Agrawal N, Majumdar A, Deshpande N, Jhulka S, Minakshisundaram S, Chawla M, Lodha S, Maheshwari A, Makkar BM, Rao S, Shah P, Ghosh R, Mohanasundaram S, Menon S, Chodankar D, Kanade V, Trivedi C (2020). Suboptimal glycemic control among subjects with diabetes mellitus in India: a subset analysis of cross-sectional wave-7 (2016) data from the international diabetes management practices study (IDMPS). Ther Adv Endocrinol Metab.

[3] Wang L, Peng W, Zhao Z, Zhang M, Shi Z, Song Z, Zhang X, Li C, Huang Z, Sun X, Wang L, Zhou M, Wu J, Wang Y (2021). Prevalence and treatment of diabetes in China, 2013-2018. JAMA.

[4] Silva JAD, Souza ECF, Echazú Böschemeier AG, Costa C, Bezerra HS, Feitosa E (2018). Diagnosis of diabetes mellitus and living with a chronic condition: participatory study. BMC Public Health.

[5] Jia W, Weng J, Zhu D, Ji L, Lu J, Zhou Z, Zou D, Guo L, Ji Q, Chen L, Chen L, Dou J, Guo X, Kuang H, Li L, Li Q, Li X, Liu J, Ran X, Shi L, Song G, Xiao X, Yang L, Zhao Z (2019). Standards of medical care for type 2 diabetes in China 2019. Diabetes Metab Res Rev.

[6] Ji L, Kang ES, Dong X, Li L, Yuan G, Shang S, Niemoeller E (2020). Efficacy and safety of insulin glargine 300 U/mL versus insulin glargine 100 U/mL in Asia Pacific insulin-naïve people with type 2 diabetes: the EDITION AP randomized controlled trial. Diabetes Obes Metab.

[7] Zhang P, Chen M, Zhang H, Luo Y, Zhu D, Li X, Ji J, Wang D, Duolikun N, Ji L (2022). Effectiveness and safety of basal insulin therapy in type 2 diabetes mellitus patients with or without metformin observed in a national cohort in China. BMC Endocr Disord.

[8] Kaufman N (2019). Digital therapeutics: leading the way to improved outcomes for people with diabetes. Diabetes Spectr.

[9] Adhikari M, Devkota HR, Cesuroglu T (2021). Barriers to and facilitators of diabetes self-management practices in Rupandehi, Nepal—multiple stakeholders' perspective. BMC Public Health.

[10] Agarwal S, Simmonds I, Myers AK (2022). The use of diabetes technology to address inequity in health outcomes: limitations and opportunities. Curr Diab Rep.

[11] Iregbu S, Spiers J, Duggleby W, Salami B, Schick-Makaroff K (2023). Nigerian health care providers and diabetes self-management support: their perspectives and practices. Qual Health Res.

[12] Wang C, Lee C, Shin H (2023). Digital therapeutics from bench to bedside. NPJ Digit Med.

[13] Holmes D (2017). Pharmacotherapy: a smarter way to treat obesity. Nat Rev Endocrinol.

[14] Hou C, Carter B, Hewitt J, Francisa T, Mayor S (2016). Do mobile phone applications improve glycemic control (HbA1c) in the self-management of diabetes? A systematic review, meta-analysis, and GRADE of 14 randomized trials. Diabetes Care.

[15] Sepah SC, Jiang L, Ellis RJ, McDermott K, Peters AL (2017). Engagement and outcomes in a digital diabetes prevention program: 3-year update. BMJ Open Diabetes Res Care.

[16] Lin J, Li X, Jiang S, Ma X, Yang Y, Zhou Z (2020). Utilizing technology-enabled intervention to improve blood glucose self-management outcome in type 2 diabetic patients initiated on insulin therapy: a retrospective real-world study. Int J Endocrinol.

[17] Böhm AK, Jensen ML, Sørensen MR, Stargardt T (2020). Real-world evidence of user engagement with mobile health for diabetes management: longitudinal observational study. JMIR Mhealth Uhealth.

[18] Buch A, Yeshurun S, Cramer T, Baumann A, Sencelsky Y, Zelber Sagi S, Serebro M, Greenman Y, Mor M, Eldor R (2023). The effects of metabolism tracker device (Lumen) usage on metabolic control in adults with prediabetes: pilot clinical trial. Obes Facts.

[19] Lorig K, Ritter PL, Turner RM, English K, Laurent DD, Greenberg J (2016). Benefits of diabetes self-management for health plan members: a 6-month translation study. J Med Internet Res.

[20] Hermanns N, Ehrmann D, Finke-Groene K, Krichbaum M, Roos T, Haak T, Freckmann G, Kulzer B (2023). Use of smartphone application versus written titration charts for basal insulin titration in adults with type 2 diabetes and suboptimal glycaemic control (My Dose Coach): multicentre, open-label, parallel, randomised controlled trial. Lancet Reg Health Eur.

[21] Ji L, Zhang P, Zhu D, Li X, Ji J, Lu J, Guo X, Jia W, Weng J, Wu Y, Yang W, Zou D, Zhou Z, Pan C, Gao Y, Garg SK (2017). Observational registry of basal insulin treatment (ORBIT) in patients with type 2 diabetes uncontrolled with oral antihyperglycaemic drugs: real-life use of basal insulin in China. Diabetes Obes Metab.

[22] Tsai ST, Pathan F, Ji L, Yeung VT, Chadha M, Suastika K, Son HS, Tan KEK, Benjasuratwong Y, Nguyen TK, Iqbal F (2011). First insulinization with basal insulin in patients with type 2 diabetes in a real-world setting in Asia. J Diabetes.

[23] Nathan DM, Buse JB, Davidson MB, Ferrannini E, Holman RR, Sherwin R, Zinman B (2009). Medical management of hyperglycemia in type 2 diabetes: a consensus algorithm for the initiation and adjustment of therapy: a consensus statement of the American Diabetes Association and the European Association for the Study of Diabetes. Diabetes Care.

[24] Goodall G, Sarpong EM, Hayes C, Valentine WJ (2009). The consequences of delaying insulin initiation in UK type 2 diabetes patients failing oral hyperglycaemic agents: a modelling study. BMC Endocr Disord.

[25] Kim SG, Kim NH, Ku BJ, Shon HS, Kim DM, Park TS, Kim Y, Kim IJ, Choi DS (2017). Delay of insulin initiation in patients with type 2 diabetes mellitus inadequately controlled with oral hypoglycemic agents (analysis of patient- and physician-related factors): a prospective observational DIPP-FACTOR study in Korea. J Diabetes Investig.

[26] Chen P, Ma X, Chen H, Wang K, Zhou L (2020). Delays in insulin initiation among patients with type 2 diabetes mellitus in southeast China: a retrospective, real-world study. Diabetes Metab Syndr Obes.

[27] Davies MJ, D'Alessio DA, Fradkin J, Kernan WN, Mathieu C, Mingrone G, Rossing P, Tsapas A, Wexler DJ, Buse JB (2018). Management of hyperglycemia in type 2 diabetes, 2018. A consensus report by the American Diabetes Association (ADA) and the European Association for the Study of Diabetes (EASD). Diabetes Care.

[28] American Diabetes Association (2018). 6. Glycemic targets standards of medical care in diabetes—2018. Diabetes Care.

[29] Garber AJ, Abrahamson MJ, Barzilay JI, Blonde L, Bloomgarden ZT, Bush MA, Dagogo-Jack S, DeFronzo RA, Einhorn D, Fonseca VA, Garber JR, Garvey WT, Grunberger G, Handelsman Y, Hirsch IB, Jellinger PS, McGill JB, Mechanick JI, Rosenblit PD, Umpierrez GE (2018). Consensus statement by the American Association of Clinical Endocrinologists and American College of Endocrinology on the comprehensive type 2 diabetes management algorithm—2018 executive summary. Endocr Prac.

[30] Yang W, Ma J, Yuan G, Li L, Zhang M, Lu Y (2019). Determining the optimal fasting glucose target for patients with type 2 diabetes: results of the multicentre, open-label, randomized-controlled FPG GOAL trial. Diabetes Obes Metab.

[31] Yuan L, Li F, Zhou Y, Sun R, Gao G, Zhang Q, Tang Y, Dai L, Wu J, Ma J (2021). Fasting glucose of 6.1 mmol/L as a possible optimal target for type 2 diabetic patients with insulin glargine: a randomized clinical trial. J Diabetes Res.

[32] Shi G, Zhu N, Qiu L, Yan H, Zeng L, Wang D, Dang S, Zhaoqing L, Kang Y, Chen T, Li C (2021). Impact of the 2020 China Diabetes Society guideline on the prevalence of diabetes mellitus and eligibility for antidiabetic treatment in China. Int J Gen Med.

