# Supplementary material for: Effectiveness and Safety of the TRIO Optimal Health Management Program in Patients With Type 2 Diabetes Mellitus Initiating Basal Insulin Therapy: Prospective Observational Real-World Study
Source: J Med Internet Res. 2025 Jan 13;27:e67554. doi: 10.2196/67554 (PMC11773279; doi:10.2196/67554)
Supplement: Multimedia Appendix 1 [file jmir_v27i1e67554_app1.docx]

| Table S1 Baseline characteristics of patients who remained at month 3 and those who were lost to follow-up of the TRIO optimal health management program. | | |
| --- | --- | --- |
| Baseline characteristics | Remained at month 3  (*N*=118,134) | Lost to follow-up  (*N*=81,297) |
| Age (years) | 57.2 (12.4) | 57.4 (12.5) |
| Gender |  |  |
| Male | 46442 (57.3) | 67424 (57.1) |
| Female | 34627 (42.7) | 50710 (42.9) |
| BMI^a^ (kg/m^2^) | 24.8 (3.5) | 24.8 (3.4) |
| Duration of diabetes (years) | 7.4 (6.8) | 7.0 (6.4) |
| SBP^b^ (mmHg) | 131.9 (16.0) | 131.6 (16.4) |
| DBP^c^ (mmHg) | 79.5 (10.1) | 80.0 (10.7) |
| Triglycerides (mmol/L) | 2.3 (2.0) | 2.3 (2.2) |
| Total cholesterol (mmol/L) | 4.7 (1.5) | 4.7 (1.5) |
| LDL^d^ (mmol/L) | 2.8 (1.1) | 2.8 (1.1) |
| Insulin dose (U/day) | 15.7 (5.9) | 16.1 (5.9) |
| Insulin dose (U/kg/day) | 0.23 (0.09) | 0.24 (0.09) |
| eGFR^e^ (ml/min/1.73m^2^) |  |  |
| <60 | 3467 (21.9) | 1485 (23.2) |
| [60,90) | 4223 (26.7) | 1650 (25.8) |
| ≥90 | 8113 (51.3) | 3253 (50.9) |
| Baseline HbA1c^f^ (%) | 9.6 (2.0) | 9.6 (2.0) |
| Baseline FPG^g^ (mmol/L) | 9.5 (3.3) | 9.5 (3.3) |
| Baseline PPG^h^ (mmol/L) | 12.8 (4.2) | 12.7 (4.2) |
| Regimen |  |  |
| BI^i^ alone ± OAD^j^ | 92459 (78.3) | 67471 (83.5) |
| BI + prandial insulin ± OAD | 24342 (20.6) | 12619 (15.6) |
| BI + GLP-1 RA^k^ ± OAD | 1329 (1.1) | 749 (0.9) |
| Education |  |  |
| Primary school | 21662 (21.4) | 17260 (25.7) |
| Secondary school | 57230 (56.6) | 35188 (52.5) |
| College and university | 22302 (22.0) | 14617 (21.8) |
| Income (RMB) |  |  |
| <3500 | 32958 (41.4) | 22037 (45.8) |
| 3500-6999 | 40129 (50.4) | 23552 (48.9) |
| 7000-10000 | 5608 (7.0) | 2173 (4.5) |
| >10000 | 985 (1.2) | 404 (0.8) |
| Comorbidity |  |  |
| Hypertension | 35395 (30.5) | 20242 (25.3) |
| Hyperlipemia | 19879 (17.1) | 12361 (15.5) |
| Left ventricular hypertrophy | 126 (0.1) | 76 (0.1) |
| Atrial fibrillation | 176 (0.2) | 117 (0.2) |
| Complication |  |  |
| Stroke | 3948 (3.4) | 1972 (2.5) |
| Coronary heart disease | 10114 (8.7) | 6143 (7.7) |
| Diabetic nephropathy | 9206 (7.9) | 5269 (6.6) |
| Diabetic retinopathy | 14964 (12.9) | 8423 (10.53) |
| Diabetic foot | 2599 (2.2) | 1317 (1.7) |
| Peripheral neuropathy | 39446 (34.0) | 23699 (29.6) |
| Lower extremity angiopathy | 8685 (7.5) | 4760 (6.0) |

^a^BMI: body mass index.

^b^SBP: systolic blood pressure.

^c^DBP: diastolic blood pressure.

^d^LDL: low-density lipoprotein.

^e^eGFR: estimated glomerular filtration rate.

^f^HbA1c: glycated hemoglobin.

^g^FPG: fasting plasma glucose.

^h^PPG: postprandial glucose.

^i^BI: basal insulin.

^j^OADs: oral antidiabetic drugs.

^k^GLP-1RA: glucagon-like peptide-1 receptor agonists.

| Table S2 Concomitant OADs and initial BI regimens in the TRIO optimal health management program. | | | |
| --- | --- | --- | --- |
|  | Outpatient | Inpatient | All |
| No. of concomitant OAD^a^s |  |  |  |
| 0 | 9451 (19.4) | 20028 (22.0) | 29479 (21.1) |
| 1 | 21950 (45.1) | 35292 (38.8) | 57242 (41.0) |
| 2 | 13651 (28.0) | 25822 (28.4) | 39473 (28.2) |
| ≥3 | 3644 (7.5) | 9901 (10.9) | 13545 (9.7) |
| Metformin | 24174 (49.6) | 44853 (49.3) | 69027 (49.4) |
| α-glucosidase | 15216 (31.2) | 31744 (34.9) | 46960 (33.6) |
| SGLT-2^b^ inhibitor | 6296 (12.9) | 16615 (18.2) | 22911 (16.4) |
| DPP-4^c^ inhibitor | 7374 (15.1) | 13348 (14.7) | 20722 (14.8) |
| Sulfonylureas | 3571 (7.3) | 3695 (4.1) | 7266 (5.2) |
| Glinides | 2319 (4.8) | 3938 (4.3) | 6257 (4.5) |
| Thiazolidinediones | 1665 (3.4) | 3839 (4.2) | 5504 (3.9) |
| BI^d^ type |  |  |  |
| Insulin glargine | 62778 (88.9) | 114553 (89.3) | 177331 (89.1) |
| Insulin determir | 1748 (2.5) | 4840 (3.8) | 6588 (3.3) |
| NPH^e^ insulin | 480 (0.7) | 286 (0.2) | 766 (0.4) |
| Insulin degludec | 2584 (3.7) | 6355 (5) | 8939 (4.5) |

^a^OADs: oral antidiabetic drugs.

^b^SGLT-2: sodium/glucose cotransporter 2.

^c^DPP-4: dipeptidyl peptidase 4.

^d^BI: basal insulin.

^e^NPH: neutral protamine hagedorn.

| Table S3 Percentage of patients reaching FBG target and experiencing hypoglycemic events at Week 1, 2, 4, 8 and 12 of the TRIO optimal health management program. | | | | | |
| --- | --- | --- | --- | --- | --- |
| Group | Week 1 | Week 2 | Week 4 | Week 8 | Week 12 |
| Percentage of patients reaching FBG^a^ target <7 mmol/L | | | | | |
| All | 30914 (54.2) | 46056 (58.5) | 44384 (60.2) | 39631 (60.4) | 37017 (61.3) |
| Dose Up | 1920 (50.27) | 6271 (54.98) | 6526 (57.24) | 6479 (56.95) | 7499 (57.37) |
| Dose Keep | 348 (58.1) | 1137 (59.75) | 1156 (61.26) | 1213 (64.49) | 1403 (62.63) |
| Dose Down | 2445 (55.8) | 7911 (60.5) | 8153 (62.69) | 8152 (62.92) | 9485 (63.34) |
| Percentage of patients experiencing hypoglycemic events (SMBG^b^ ≤3.9 mmol/L) | | | | | |
| All | 1294 (10.6) | 1036 (8.5) | 1174 (9.6) | 1039 (8.5) | 502 (4.1) |
| Dose Up | 23 (8.71) | 14 (5.3) | 20 (7.58) | 21 (7.95) | 17 (6.44) |
| Dose Keep | 4 (11.43) | 3 (8.57) | 3 (8.57) | 2 (5.71) | 6 (17.14) |
| Dose Down | 52 (13.23) | 45 (11.45) | 66 (16.79) | 52 (13.23) | 30 (7.63) |

^a^FPG: fasting blood glucose.

^b^SMBG: self-monitoring blood glucose.

Figure S1. FPG at baseline and FPG by the visit of the TRIO optimal health management program.
